# Sustainable recovery from pig slurry using ionic liquid microbial fuel cells and microalgae consortia

**DOI:** 10.1007/s00253-025-13686-w

**Published:** 2026-01-10

**Authors:** Eduardo Iniesta-López, Alfredo José Micol Blaya, Adrián Hernández Fernández, Ana Sánchez Zurano, Yolanda Garrido, Antonia Pérez de los Ríos, Francisco José Hernández Fernández

**Affiliations:** Department of Chemical Engineering, Faculty of Chemistry, University of Murcia, Campus de Espinardo, 30100 Murcia, Spain

**Keywords:** Bioelectrochemical cells, Nutrient recovery, Energy, Biomass, Algae

## Abstract

**Abstract:**

Pig slurry management has emerged as a pressing environmental challenge in the context of rapid population growth and intensified livestock production, highlighting the need for sustainable recovery technologies. While microalgae–bacteria (MB) systems offer promising opportunities for nutrient recycling, the high turbidity of raw pig slurry (PS) typically limits their direct application. This study proposes an innovative two-step treatment that combines microbial fuel cells (MFCs) with MB consortia to enhance both pollutant removal and resource recovery from raw PS with COD levels exceeding 18,000 mg·L⁻^1^. Unlike conventional designs relying on perfluorinated membranes, the MFCs employed an ionic liquid [N_8-10,8–10,8–10,1_^+^][Cl^−^] as a proton exchange medium, achieving 50% of COD removal and generating 57.27 ± 10.99 mW·m⁻^2^. The effluent was subsequently treated with MB consortia, yielding biomass productivities of 0.1 to 0.2 g·L⁻^1^·day⁻^1^, comparable to chemical fertilizer-based controls. Cell density with pre-treated and untreated pig slurry also matched control levels. In pollutant recovery, the combined microbial fuel cell and microalgae-bacteria treatment achieved up to 67% recovery of COD, over 99% of N-NH_4_^+^, and between 65 and 85% of P-PO_4_^3−^. These findings highlight the potential of integrating MFCs with MB consortia as a strategy for raw pig slurry management, t-ransforming waste into renewable energy and bioresources.

**Key points:**

• *Pig slurry is transformed into biomass and bioenergy using sustainable technologies*

• *Microalgae-bacteria consortia enhance nutrient recovery and water treatment*

• *Ionic liquid microbial fuel cells support energy generation and COD reduction*

**Graphical Abstract:**

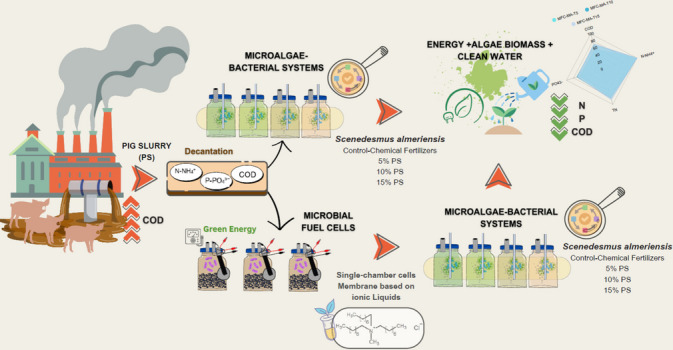

## Introduction

Global demand for livestock products, driven by population and dietary shifts, has intensified production, particularly in the European Union. Countries like Spain, Germany, and the Netherlands face significant environmental challenges due to high pig densities, hindering their ability to meet National Emission Reduction Commitments (NERC) targets for ammonia (NH_3_) reduction (Thiermann et al. 2023; Haddad et al. 2024). Current data show that these countries still need to meet their NH_3_ reduction targets for 2030 (European Environment Agency 2021). A major source of these emissions is pig slurry (PS), an organic residue rich in pollutants, including high concentrations of Chemical Oxygen Demand (COD), and valuable macronutrients like nitrogen (N) and phosphorus (P). However, its considerable water content poses challenges for both handling and transportation (Marszałek et al. 2019).

In recent years, microalgae-based technologies have been proposed for PS treatment (Min et al. 2014; Zheng et al. 2019; Sánchez-Zurano et al. 2021; Ferreira et al. 2021). This approach offers advantages over conventional methods, such as (i) lower aeration and operating costs, (ii) reduced GHG emissions through CO_2_ capturers, (iii) nutrient recovery as biomass (Acién et al. 2016; Ferreira et al. 2023). Biomass from PS can be valorized for industrial applications (Juárez et al. 2020). Microalgae are photosynthetic microorganisms that use light and CO_2_, while consuming mineral nutrients (N, P). Their oxygen (O_2_) production enables symbiosis with bacteria that oxidize organic matter. In MB systems, bacteria degrade organic matter, releasing CO_2_ and nutrients that microalgae assimilate, producing O_2_ to sustain the cycle (Posadas et al. 2017; Fallahi et al. 2021). The use of MB for PS treatment is limited by high turbidity and color from suspended solids (SS) and dissolved organic matter (DOM), which reduce light transmission and hinder microalgal photosynthesis and nutrient recycling. In aerobic processes can be directly applied when COD is below 4000 mg·L⁻^1^, whereas higher concentrations (> 10,000 mg·L⁻^1^) require pre-treatment. While coagulation/flocculation effectively mitigates these issues, it generates large amounts of sludge, posing significant treatment and handling challenges (Yu et al. 2024).

Nevertheless, anaerobic digestion (AD) remains the most mature and economically viable technology for large-scale pig slurry management, efficiently converting organic matter into biogas (mainly CH₄) that can be used as renewable energy (Nasir et al. 2012). Recent studies explore Microbial Fuel Cells (MFCs) as a complementary or alternative bioelectrochemical route, focusing on nutrient recovery and pollutant removal rather than energy production. Over the past twenty years, it has attracted considerable research interest as a strategy for COD reduction in wastewater treatment, while simultaneously addressing the global energy crisis (Bird et al. 2022). This approach enables electricity generation through the metabolic activity of electroactive microorganisms naturally present in wastewater (Hernández-Fernández et al. 2015). As an anaerobic treatment method, MFCs are capable of recovering energy from waste streams under ambient and low-temperature conditions. Their operation relies on electroactive bacteria that oxidize organic substrates within a bioelectrochemical system, where an anaerobic anode is coupled to an aerobic cathode, typically separated by a proton exchange membrane (PEM). Electrons flow through an external circuit to a high-potential acceptor like O_2_. Using PS organic matter as fuel, MFCs remove pollutants while generating electricity (Logan 2009; Zhang et al. 2014; Pandit et al. 2017). Traditionally, perfluorinated proton exchange membranes (PEMs) have been employed in MFCs due to their excellent proton conductivity. However, their reliance on perfluoroalkyl and polyfluoroalkyl substances (PFAS) raises serious environmental and regulatory concerns. The European Chemicals Agency (ECHA) is currently evaluating the potential consequences of restricting nearly 10,000 PFAS compounds under Regulation (EU) No. 1907/2006 (REACH). Such restrictions would directly challenge the continued use of perfluorinated PEMs, making the identification of potential alternatives not only desirable but imperative. In this context, the development of non-perfluorinated PEMs emerges as a critical research priority, driven by the environmental and regulatory concerns surrounding traditional PFAS-based membranes. Ionic Liquid (IL)-based membranes offer a promising alternative, providing low cost, enhanced stability, and improved environmental compatibility, thus advancing the long-term viability of MFC technology (Koók et al. 2017; Hernández-Fernández et al. 2023). Previous studies have successfully applied the ILs methyltrioctylammonium chloride [N_8,8,8,1_^+^][Cl^−^] and methylalkylammonium chloride [N_8–10,8–10,8–10,1_^+^][Cl^−^] as a polymer inclusion membranes (PIMs) in a single-chamber MFC, employing wastewater from an oil factory (COD content of 430 mg·L^−1^) (Salar-García et al. 2015), raw industrial wastewater (COD load of 992 mg·L^−1^) (Ortiz-Martínez et al. 2015), wastewater from an industrial factory (COD content 1200 mg·L^−1^) (Salar-García et al. 2016), and PS with concentrations above 2000 mg·L^−1^ (Hernández-Fernández et al. 2024; Iniesta-López et al. 2025). Crucially, the performance of these IL-PIMs in MFCs has not yet been validated with high-concentration wastewater, specifically those exceeding 10,000 mg·L^−1^ COD. This gap represents a significant barrier to their practical application in treating raw PS from intensive livestock farming.

At present, two main strategies have been explored to couple MFCs with microalgal (MB) cultivation. The first approach directs partially treated effluent from the MFC’s anode into a MB reactor to enhance nutrient removal. The second integrates microalgae directly at the cathode, where they function as biocatalysts to increase O_2_ availability and, consequently, electricity generation. The first one has shown greater effectiveness, improved wastewater treatment, addressing MFC technology limitations, and generating algal biomass for industrial applications. In this configuration, both the MFC and microalgal photobioreactor systems operate independently (Jiang et al. 2013; Chiranjeevi and Patil 2020). Therefore, a preliminary treatment of PS in MFCs would reduce the content of SS and DOM in a sustainable way while green electricity is generated. This pre-treated PS, could then be processed in an MB system to recover the C, N, and P present in the form of valuable biomass. This operational process has already been successfully addressed in previous studies using PS with concentrations above 2000 mg·L^−1^ in the anode, and diluted PS in the cathode, separated by a [N_8–10,8–10,8–10,1_^+^][Cl^−^] membrane and polyvinyl chloride (PVC) (Iniesta-López et al. 2025). However, it has not yet been validated using raw PS directly obtained from an industrial livestock facility.

Therefore, the implementation of an MFC pre-treatment, utilizing PIMs made from IL [N_8–10,8–10,8–10,1_^+^][Cl^−^] is expected to enhance the performance of the MB system for treating high-concentration raw PS. Accordingly, the overall objective of this work is threefold: (i) to systematically compare the nutrient removal efficiencies achieved by treating raw, high-concentration PS from a commercial farm using two distinct approaches, direct MB treatment versus a combined two-stage MFCMB approach; (ii) to evaluate the performance of the MFC developed with a non-perfluorinated PIM made from IL [N_8–10,8–10,8–10,1_^+^][Cl^−^] and polyvinyl chloride (PVC) in terms of electricity production and COD reduction using the raw PS as fuel; and finally, (iii) to quantify the resource recovery potential of the MB consortia by assessing the biomass productivity (using *S. almeriensis*) and the subsequent recovery of nutrient from both the pre-treated MFC effluent and untreated PS.

## Material and methods

### Microorganisms and growth media

The microalga *Scenedesmus almeriensis*, obtained from the culture collection of the Desalination and Photosynthesis Research Group at the University of Almería, was selected for the experimental assays. Preparation of the inoculum required a mineral growth medium, which was formulated using chemical fertilizers: 0.9 g·L⁻^1^ of NaNO₃, 0.18 g·L⁻^1^ of MgSO₄ · 7H₂O, 0.14 g·L⁻^1^ of KH₂PO₄, and 0.02 g·L⁻^1^ of Karentol. PS from Sangonera La Verde Farm in Murcia, Spain, was used, and PS was also used as the inoculum to establish an anaerobic bacterial community. After primary sedimentation, the composition of PS is outlined in Table 1.

**Table 1:** Composition of the PS after decantation primary treatment and MFCs treatment

Parameter	Decanted PS	MFCs
COD (mg·L^−1^)	18,030 ± 42.43	7890 ± 69.28
N-NH_4_^+^ (mg·L^−1^)	1825 ± 80.1	1975 ± 28.9
N-NO_2_^−^ (mg·L^−1^)	0.6 ± 0.4	1.4 ± 0.5
N-NO_3_^−^ (mg·L^−1^)	< LOD	< LOD
TN (mg·L^−1^)	2400 ± 247.49	2350 ± 177.95
P-PO_4_^−3^ (mg·L^−1^)	19.28 ± 0.53	18.15 ± 0.93
SS (g·L^−1^)	10.19 ± 0.69	1.96 ± 0.3

^*^PS, Pig Slurry; < *LOD*, Limit of detection

**Fig. 1:**
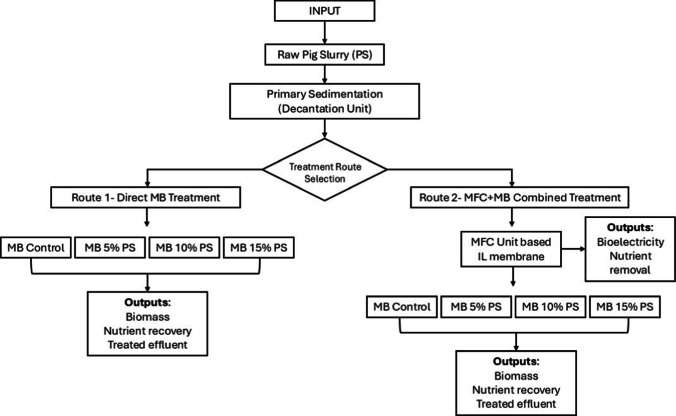
Process flow overview

After decanting the PS, two tests were performed. First, the PS was treated using the MFCs, and the resulting pre-treated water was then used to prepare the culture medium for *S. almeriensis*. For this, the pretreated PS with MFCs was diluted to 5% (5% PS), 10% (10% PS), and 15% (15% PS) with distilled water. Additionally, another assay was performed, where the PS after primary decantation was directly diluted to 5% (5% PS), 10% (10% PS), and 15% (15% PS) with distilled water. These culture media were used to evaluate the growth of *S. almeriensis*. In addition, a control treatment was included using the chemical fertilizer medium previously described (Control). The initial concentrations of the culture media employed in both experiments are detailed in Table 2. The flow overview process is included in Fig. 1.

**Table 2:** Composition of the culture medium used for treatment with MB when performed as a single treatment and combined with MFCs

Parameter	Control	5% PS		10% PS		15% PS	
		MB	MFCs + MB	MB	MFCs + MB	MB	MFCs + MB
COD (mg·L^−1^)	70.10 ± 6.79	925.70 ± 1.55	419 ± 4.24	1827.20 ± 3.68	813 ± 8.49	2728.70 ± 5.80	1207.50 ± 12.02
N-NH_4_^+^ (mg·L^−1^)	0.01 ± 0.0	88.4 ± 0.1	90 ± 0.00	189.1 ± 0.1	190.5 ± 2.1	280.3 ± 0.1	284.0 ± 5.7
N-NO_2_^−^ (mg·L^−1^)	0.02 ± 0.01	1.33 ± 0.021	0.34 ± 0.001	1.33 ± 0.21	0.33 ± 0.003	1.33 ± 0.21	0.33 ± 0.00
N-NO_3_^−^ (mg·L^−1^)	126.76 ± 0.45	6.25 ± 0.03	8.05 ± 0.120	12.50 ± 0.05	7.97 ± 0.37	18.74 ± 0.08	8.22 ± 0.002
TN (mg·L^−1^)	149 ± 13.44	134.25 ± 10.96	112.50 ± 3.54	235.50 ± 23.33	215 ± 0.00	336.75 ± 35.71	307.50 ± 10.61
P-PO_4_^−3^ (mg·L^−1^)	27.56 ± 0.47	3.24 ± 0.016	1.85 ± 0.07	4.38 ± 0.01	2.85 ± 0.07	5.52 ± 0.01	4.15 ± 0.07
SS (g·L^−1^)	0.27 ± 0.00	0.52 ± 0.01	0.22 ± 0.01	0.88 ± 0.07	0.3 ± 0.07	1.47 ± 0.03	0.47 ± 0.11

^*^PS, Pig Slurry; *MB*, microalgae-bacteria; *MFCs*, Microbial Fuel Cells

### Preparation of membrane electrode assembly (MEA) using ionic liquids

The MEA was constructed employing PIM as PEM. The ionic liquid selected was Methylalkylammonium chloride [N_8–10,8–10,8–10,1_^+^][Cl^−^] ([MTAA^+^][Cl^−^]) (Sigma-Aldrich) with polyvinyl chloride (PVC) from Fluka (Sigma-Aldrich) used as the polymer matrix. These membranes were fabricated via the casting technique, comprising 70% w/w [N_8–10,8–10,8–10,1_^+^][Cl^−^] and 30% w/w PVC as described (Iniesta-López et al. 2025). A total mass of 300 mg of membrane was obtained, composed of 70 wt% [N_8–10,8–10,8–10,1_^+^][Cl^−^] (210 mg) and 30 wt% PVC (90 mg). The mixture was dissolved in 1.5–3 mL of tetrahydrofuran (THF, PanReac, ApplyChem) under magnetic stirring until complete homogenization. The homogeneous solution was cast within a borosilicate ring (3.5 cm diameter, Anorsa X-8374) placed on a glass plate and left to evaporate for 24 h at room temperature. The resulting membrane had an active area of 18.934 cm^2^ and an average thickness of approximately 120 ± 10 µm, as measured using a micrometer.

Following membrane preparation, a platinum catalyst was applied using an aerosol technique, drawing on previous successful applications in conventional cathode catalyst layers. Each membrane was coated to reach a predetermined platinum loading, with adjustments made according to catalyst purity. The catalyst ink was prepared with a total Pt loading of 0.5 mg·cm⁻^2^, corresponding to 9.665 mg of Pt per membrane. For the catalyst ink, isopropanol was used as the solvent, added in relation to the membrane’s active surface area at a ratio equivalent to 0.4 ml·cm^−2^, giving a total volume of 7.732 mL for the membrane area.

After preparation, the samples were placed in an ultrasonic bath for 2 h to achieve better homogenization. The application of the catalyst was performed with an airbrush system connected to a compressor (Iwata, Japan), maintaining sufficient distance to minimize the risk of damaging the membrane surface. To avoid ink pooling, alternating spray and drying cycles were employed, supported by an infrared lamp to accelerate solvent evaporation. Finally, the membranes were left to dry on petri dishes overnight, and their mass was recorded the following day to verify successful ink deposition, as confirmed by the observed weight increase (Iniesta-López et al. 2024).

### MFCs treatment

MFC experiments were carried out in a single-chamber reactor with a total volume of 250 mL. The anode was assembled using one graphite rod together with 100 g of granular graphite (3–5 mm in diameter), plus an additional graphite rod of 3.18 mm in diameter. A membrane was placed over the reactor opening and secured with washers and an O-ring. The cathode consisted of a carbon cloth serving as the gas diffusion layer, which was positioned in direct contact with the membrane. Both electrodes were connected through a 1 kΩ resistor and held in place using two clamps. Silicone (Axton) was applied to seal the sampling port and the cathode, ensuring anaerobic conditions inside the chamber. All runs were performed in batch mode: 220 mL of PS pre-inoculated for 7 days was first added, followed by a similar PS substrate for 14 days as the fuel source. Each condition was tested in triplicate.

### Evaluation of electrochemical data

To assess the electrical output of the fuel, both the polarization curve and the internal resistance were determined. For each microbial fuel cell, a polarization test was carried out to evaluate its electrical behavior. This test consisted of varying the external resistance using a resistor box, which modified the current in the circuit and allowed the corresponding voltage at each resistance point to be recorded.

Voltage measurements were continuously monitored using a PicoLog data logger (PicoTechnology, UK), configured to capture data every 3 min and connected directly to a computer for real-time tracking. To ensure measurement accuracy, voltage values were also manually verified at intervals with a DVM891 digital multimeter (HQ Power, Berlin, Germany). A variable resistor box set to different resistances (5.77 MΩ, 953 kΩ, 486 kΩ, 96.5 kΩ, 50 kΩ, 11 kΩ, 6 kΩ, 1.1 kΩ, 561 Ω, 94.5 Ω, and 1.5 Ω) was used to measure the polarization after 240 h of operation. Voltage was recorded once the cell stabilized at a near-steady state under each resistor setting, which generally took around one minute. To improve accuracy, each measurement was repeated three times, with the average value reported. The internal resistance R_int_(Ω) was calculated using Eq. (1):

1 $${{{R}}}_{{{i}}{{n}}{{t}}}({\Omega })=\frac{{{O}}{{C}}{{V}}{ }({{V}})}{{{I}}{ }({{A}})}-{{{R}}}_{{{e}}{{x}}{{t}}{ }}({\Omega })$$

where R_ext_ (Ω) is the external resistance at maximum power, I is the current density at maximum power and OCV is the open circuit voltage.

### Lab-scale photobioreactors

To assess nutrient recovery from PS, laboratory-scale photobioreactors were employed under conditions both with and without prior treatment by MFCs, incorporating continuous aeration and without pH regulation. The system consisted of bubble column photobioreactors with a total volume of 0.25 L (6.5 cm in diameter and 14 cm in height), and a working volume of 0.20 L (Schott Duran, Germany). Aeration was maintained at a constant flow rate of 0.25 L·min^−1^, operating continuously over 24 h.

Room temperature was regulated to maintain the reactors at 25 ± 1 °C. Illumination was provided by two 44 W LED lamps placed 3 cm away from the reactor surface, delivering an average light intensity of 233.96 ± 78.32 μmol·m^−2^·s^−1^.The photoperiod was 16 h light/8 h dark.

The photobioreactors were inoculated with the *S.almeriensis* at an initial cellular density of approximately 1.4·10^6^ cell·mL^−1^. The experiments were carried out in batch mode for 12 days. Three identical reactors were used in the trials, each serving as an independent experimental unit.

### Growth monitoring

Cell quantification was carried out at the beginning, halfway through, and at the conclusion of the experiment by means of optical microscopy and a Neubauer counting chamber. This allowed monitoring of cell proliferation and the calculation of cell concentration. The cell density (C) was calculated according to Eq. (2), in which N corresponds to the mean number of cells counted within five small squares of the central grid, 4 × 10⁻⁶ corresponds to the sample volume in cm^3^ (mL) divided by the area of the small squares (0.004 mm^3^), and D is the dilution factor.

2 $${\varvec{C}}=\frac{\boldsymbol{ }{\varvec{N}}}{4\times {10}^{-6}}\times {\varvec{D}}$$

Biomass concentration was determined by measuring the dry weight. For this, 20 mL of the culture was filtered through 0.5 μm Whatman GF-F glass fiber filters and then dried in an oven at 105 °C for 24 h. Biomass productivity was calculated based on the initial and final biomass concentrations and the total duration of the experiment.

### Analytical determinations

COD was measured using established photometric methods on a SpectroQuant Prove 300 spectrophotometer (Merck Millipore), with samples prepped in a TR 420 Thermoreactor (Merck Millipore) for accuracy. Also, Total Nitrogen (TN) concentrations was determined through established photometric methods on a SpectroQuant Prove 300 spectrophotometer (Merck Millipore). Phosphate (PO_4_^3^⁻) levels were tested with 114,729 Supelco phosphate cuvettes (Sigma-Aldrich®), following APHA 4500-P E and DIN EN ISO 6878 standards. For Nitrite (NO_2_^−^) and Nitrate (NO_3_^−^) quantification, colorimetric methods were employed. NO_2_^−^ measured using the Griess reagent technique, which forms a red or pink complex upon reaction with sulfanilic acid and N-(1-naphthyl)-ethylenediamine, and is detected at 540 nm. NO_3_^−^ levels were determined via High-Performance Liquid Chromatography (HPLC) using an Agilent Technologies system, with a mobile phase consisting of 20% methanol and 0.01 M octylamine adjusted to pH 6.5. Ammonium (NH_4_^+^) concentrations were assessed using designated cuvettes and protocols provided by Sigma-Aldrich®, following the ISO 23695 standard. Suspended Solids (SS) were measured by filtering samples through Whatman GF/F glass fiber filters (0.5-µm pore size). The filters were subsequently dried at 105 °C, desiccated and weighed.

### Analysis of statistical data

An Analysis of Variance (ANOVA) was performed to assess differences among the treatments, using a significance level of *p* < 0.05. When statistically significant differences were identified, Tukey’s Honest Significant Difference (HSD) test was used for post hoc comparisons. All statistical analyses were conducted using IBM SPSS Statistics software version 28.0.1.1 (14) for Windows (IBM Corporation, Armonk, New York, USA).

## Results

In the development of this study, the treatment of PS diluted to 5, 10, and 15% was evaluated using a sole MB consortium-based treatment. Additionally, these results were compared with a combined MFC-MB treatment, in which both systems operate independently, with the output from the MFC (pre-diluted to 5, 10, and 15%) serving as the input for the MB consortium system.

### Bio-electricity generation by MFCs

**Fig. 2:**
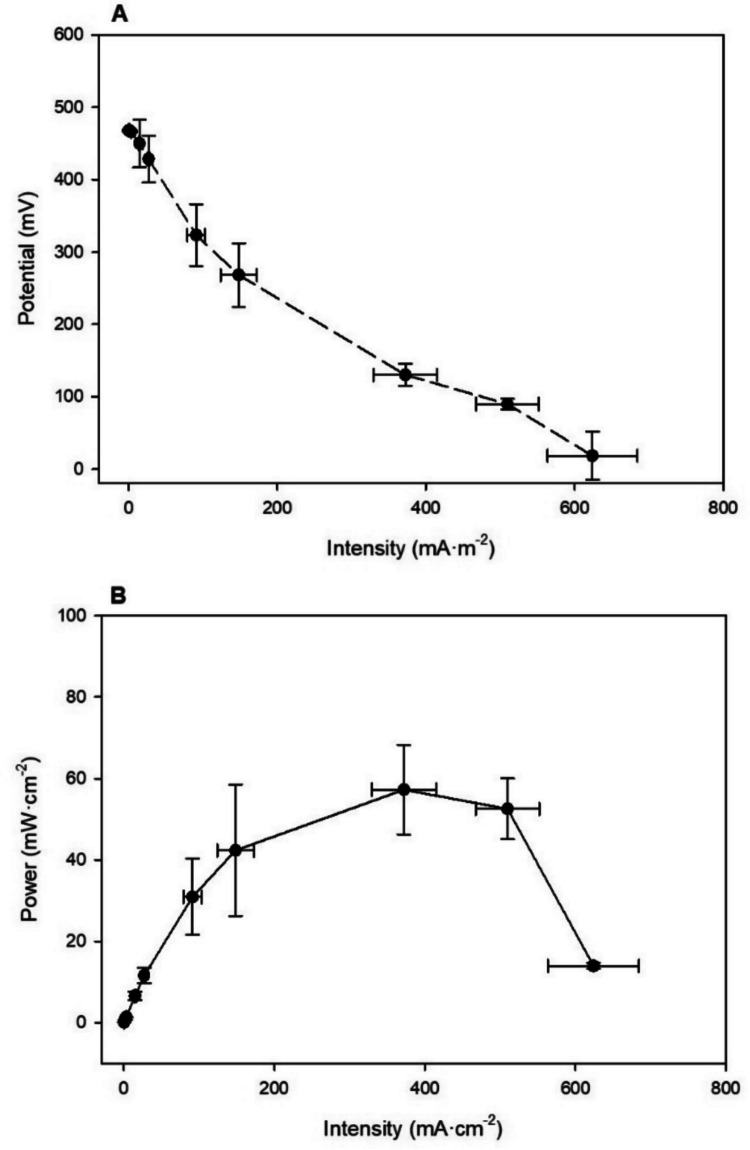
Electricity generation by MFCs using pig slurry as fuel. Polarization curve 7 days after the start of the test with calculations normalized according to the exposed cathode surface (**A**) and Polari-zation curve 7 days after the start of the test with calculations normalized according to the exposed cathode surface (**B**). (*n* = 3). Data are presented as mean values of triplicate samples ± SD

The open circuit voltage (OCV), representing infinite resistance (current density = 0), was measured at 468 ± 1.06 mV. On the other hand, the power curve, representing power as a function of current, was obtained on the final day of the experiment, as shown in Fig. 2. The maximum average power recorded was 57.27 ± 10.99 mW·m⁻^2^. Furthermore, by performing the polarization test, it was also possible to determine the external resistance at which maximum power was achieved, which was 1113 Ω.

This experiment also measured internal resistance, which is linked to diffusion constraints and structural impediments inside the MFCs, leading to energy inefficiencies. This parameter was calculated using Eq. (1), yielding an average value of 2060.85 ± 714 Ω.

### Nutrients removal by MFCs

**Fig. 3:**
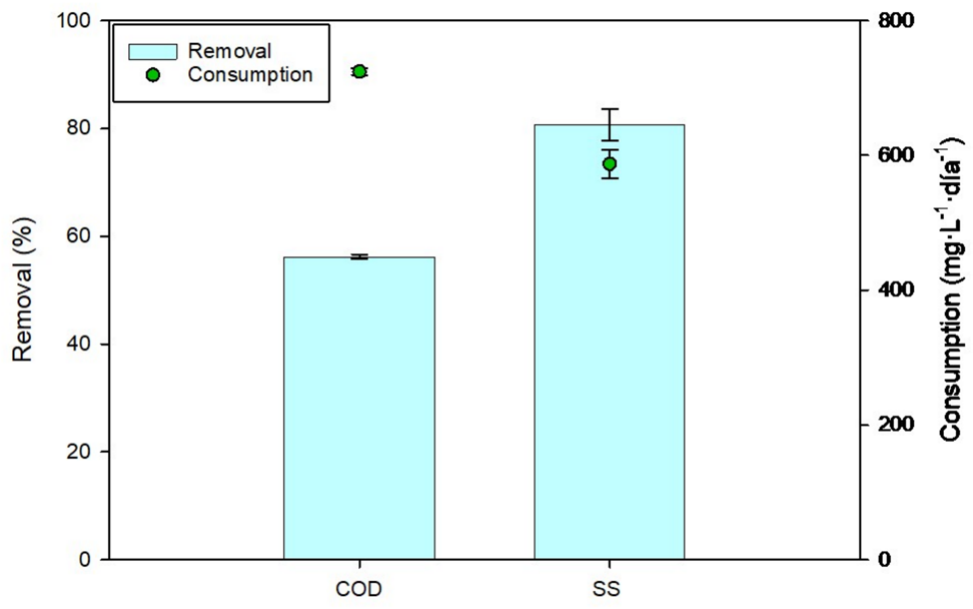
Elimination performance of MFCs using PS as fuel (*n* = 3). Data are presented as mean values of triplicate samples ± SD

Figure 3 shows both the removal efficiency and the consumption of nutrients by MFCs. As observed, the MFCs were only able to remove COD and SS. For the remaining pollutants, no statistically significant difference was observed between the initial and final concentrations. The removal of organic matter was determined based on COD. Starting from an average initial COD of 18,030 ± 42.43 mg·L⁻^1^, 56.24 ± 0.38% was removed, representing a reduction of more than half of the initial COD. SS showed the highest reduction, with an 80.74 ± 2.93% decrease, demonstrating the MFCs’ capacity to reduce suspended particles, with a consumption rate of 587.50 ± 21.29 mg·L⁻^1^·day⁻^1^.

### Microalgae-bacteria production

**Fig. 4:**
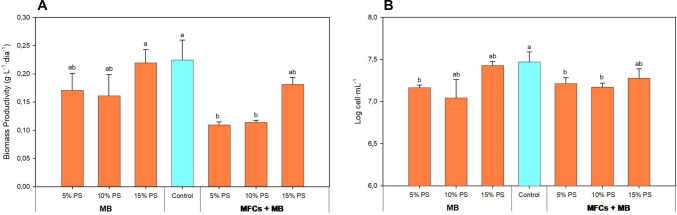
Biomass productivity (**A**) and cell density (**B**) obtained during MB treatment and MFCs + MB treatment. (*n* = 3). Data are presented as mean values of triplicate samples ± SD. Different letters show significant differences (*p* < *0.05*)

In this work, the assessment of PS diluted at 5, 10, and 15%, along with pre-treated PS using MFCs at the same percentages, was examined regarding *S. almeriensis* productivity and cell density (Fig. 4). Regarding biomass productivity, the highest value was recorded in the Control treatment, reaching 0.225 ± 0.034 g g·L⁻^1^·day⁻^1^. For treatments using PS without MFC pre-treatment, productivity ranged from 0.162 to 0.219 g·L⁻^1^·day⁻^1^. Statistical analysis showed no significant differences in productivity between the Control and treatments with PS diluted at 5, 10, and 15% (Fig. 4A).

In the case of PS pre-treated with MFCs, productivity values ranged from 0.110 g·L⁻^1^·day⁻^1^ (5% PS) to 0.182 g·L⁻^1^·day⁻^1^ (15% PS). Although no significant differences were found between the Control and the treatment with 15% PS pre-treated with MFCs, significant differences were observed when compared to the treatments with 5 and 10% pre-treated PS (*p* < 0.05).

With respect to cell density, the highest values were obtained in the Control treatment with 7.48 ± 0.12 (Log cells·mL⁻^1^), while other treatments ranged from 7.05 to 7.43 (Fig. 4B). Statistical analysis showed that both the treatment with 15% PS and the one with 15% PS pre-treated with MFCs exhibited cell densities similar to the Control (*p* < 0.05).

### Nutrient recovery by microalgae-bacteria consortia

In this work, the nutrient recovery by MB depends on the PS used as culture media since it is important to distinguish between nutrients recovered by the MB system using diluted PS without any pre-treatment and using PS as a cultivation medium after pre-treatment with MFC systems. The pre-treatment significantly affected the COD concentration in the cultivation media (*p* < 0.05).

**Fig. 5:**
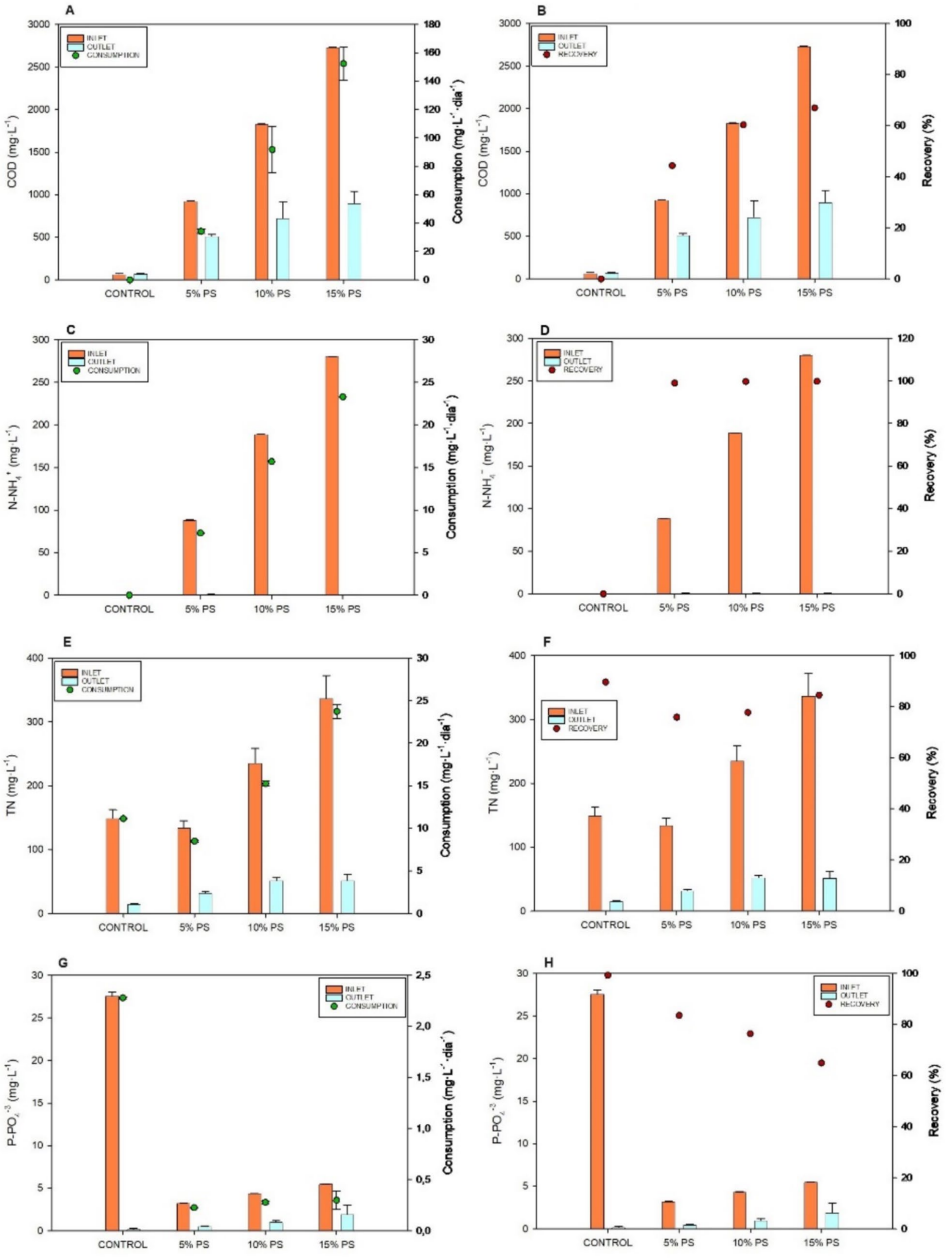
Nutrients concentration (COD, N-NH_4_^+^, TN and P-PO4^3^⁻) in the inlet and outlet culture medium along with the consumption and recovery of each one during MB growth in batch mode. Concentration of COD in the inlet and outlet culture medium and the consumption (**A**); Concentration of COD in the inlet and outlet culture medium and the recovery rate (**B**); Concentration of N-NH_4_^+^ in the inlet and outlet culture medium and the consumption (**C**); Concentration of N-NH_4_^+^ in the inlet and outlet culture medium and the recovery rate (**D**); Concentration of TN in the inlet and outlet culture medium and the consumption (**E**); Concentration of TN in the inlet and outlet culture medium and the removal rate (**F**); Concentration of P-PO4^3^⁻ in the inlet and outlet culture medium and the consumption (**G**); Concentration of P-PO4^3^⁻ in the inlet and outlet culture medium and the removal rate (**H**). (*n* = 3). Data are presented as mean values of triplicate samples ± SD

Figure 5 shows the nutrients concentration (COD, N-NH_4_^+^, TN and P-PO_4_^3−^) in the inlet and outlet culture medium along with the consumption and recovery of each one during MB growth in batch mode using diluted PS. Regarding COD, the influent concentration ranged from 925.7 to 2728.7 mg·L^−1^ using PS diluted to 5% and 15%, respectively, while the treated water concentration ranged between 515.3 and 900.7 mg·L^−1^. The consumption ranged from 34.2 to 152.3 mg·L^−1^·day^−1^ (Fig. 5A). These consumption values correspond to COD recovery rates ranging from 44 to 67% (Fig. 5B).

Regarding N, no N-NO_3_^−^ or N-NO_2_^−^ was detected in the PS used for the experiment, with the majority of N present as N-NH_4_^+^. In the prepared media, N-NH_4_^+^ ranged from 88.4 to 280.3 mg·L^−1^, while at the end of the trial, the concentration in all cases was below 1 mg·L^−1^. This corresponds to consumption rates between 7.5 and 23.3 mg·L^−1^·day^−1^, with recovery percentages exceeding 99% (Fig. 5C, D). For TN, influent values ranged between 134.3 and 336.8 mg·L^−1^, with consumption rates between 8.5 and 23.7 mg·L^−1^·day^−1^ (Fig. 5E). In the treated effluent, TN concentration ranged from 32 to 52 mg·L^−1^, being the TN recovery from 76 to 85% (Fig. 5F).

In this study, the consumption rates of P-PO_4_^3−^ ranged from 0.23 to 0.3 mg·L^−1^·day^−1^ in cultures with diluted PS, achieving over 80% removal in 5% PS (Figs. 5 G, 4H).

**Fig. 6:**
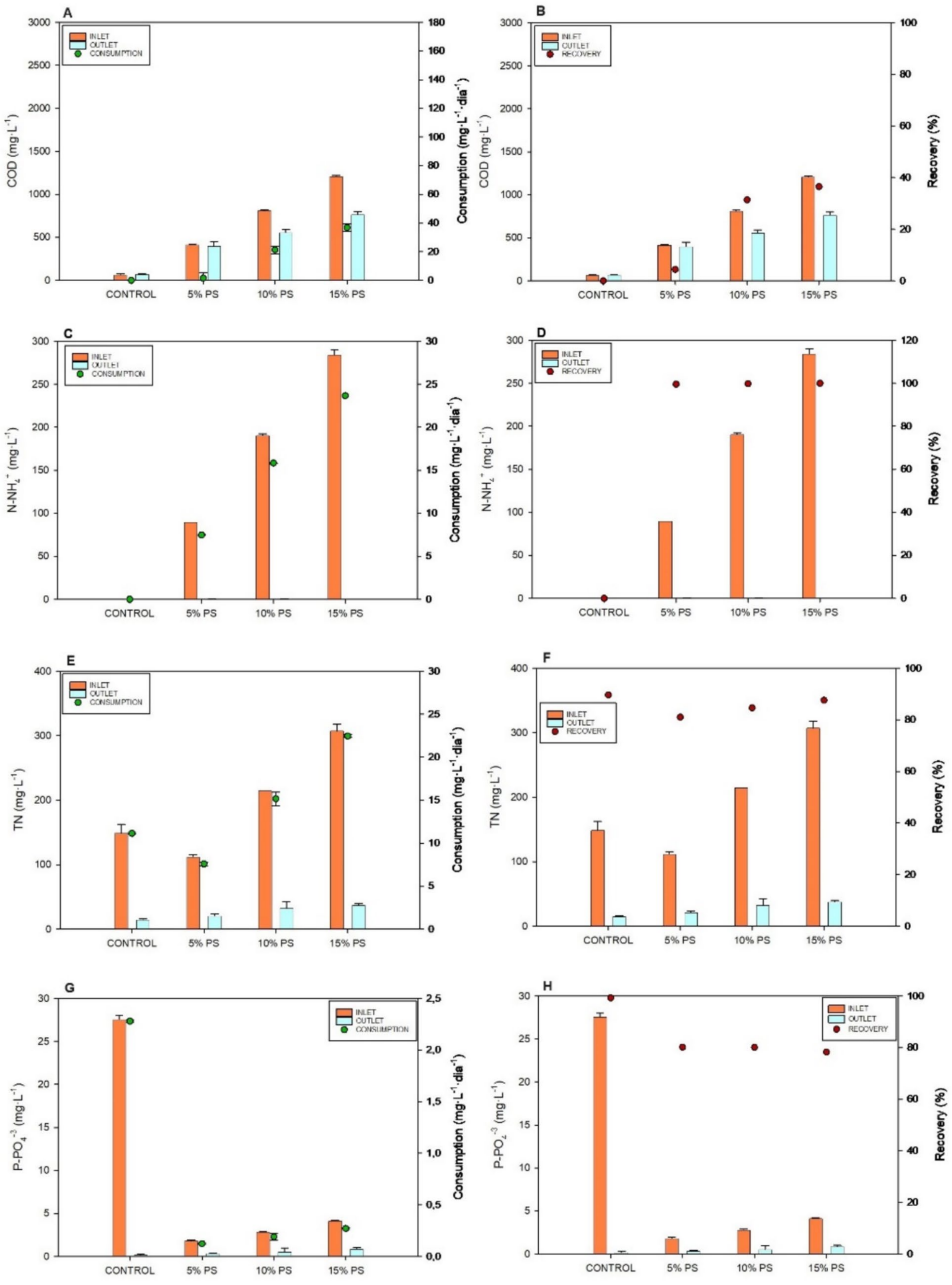
Nutrients concentration (COD, N-NH_4_^+^, TN and P-PO4^3^⁻) in the inlet and outlet culture medium along with the consumption and recovery of each one during microalgae culture growth after MFCs treatment: Concentration of COD in the inlet and outlet culture medium and the consumption (**A**); Concentration of COD in the inlet and outlet culture medium and the recovery rate (**B**); Concentration of N-NH_4_^+^ in the inlet and outlet culture medium and the consumption (**C**); Concentration of N-NH_4_^+^ in the inlet and outlet culture medium and the recovery rate (**D**); Concentration of TN in the inlet and outlet culture medium and the consumption (**E**); Concentration of TN in the inlet and outlet culture medium and the removal rate (**F**); Concentration of P-PO4^3^⁻ in the inlet and outlet culture medium and the consumption (**G**); Concentration of P-PO4^3^⁻ in the inlet and outlet culture medium and the removal rate (**H**). (*n* = 3). Data are presented as mean values of triplicate samples ± SD

After characterizing nutrient recovery from diluted PS by MB consortia, the focus shifted to evaluating nutrient consumption and recovery from PS treated with MFC technology. Figure 6 shows the nutrients concentration (COD, N-NH_4_^+^, TN and P-PO_4_^3−^) in the inlet and outlet culture medium along with the consumption and recovery of each one during MB growth in batch mode using diluted PS after MFCs treatment. MFC technology significantly reduced COD concentrations in the PS effluent while generating bioenergy. The influent COD concentration ranged from 419 to 1207 mg·L^−1^, with consumption rates between 1.5 and 36.7 mg·L^−1^·day^−1^. After combined treatment, the treated water had COD concentrations between 400.7 and 767.7 mg·L^−1^, with removal percentages ranging from 4.4% to 36.5% (Fig. 6A, B). These results indicate a notable reduction in COD compared to MB treatment alone (*p* < 0.05) (Table 2).

Related N-NH_4_^+^, It ranged from 90 to 284 mg·L^−1^, while at the end of the trial, the concentration in all cases was below 1 mg·L^−1^. This corresponds to consumption rates between 7.5 and 23.7 mg·L^−1^·day^−1^, with recovery percentages exceeding 99.7% (Fig. 6C, D). No significant differences were obtained towards the alone MB treatment. For TN, influent values ranged between 112.5 and 307.5 mg·L^−1^, with consumption rates between 7.6 and 22.5 mg·L^−1^·day^−1^. After treatment, the treated water had TN concentrations between 21.3 and 38 mg·L^−1^, with removal percentages ranging from 81.1% to 87.6% (Fig. 5E, F). These results indicate a significant reduction in TN compared to MB treatment alone (*p* < 0.05). Related to P-PO_4_^3−^, the influent concentration ranged from 1.9 to 4.2 mg·L^−1^, with consumption rates between 0.1 and 0.3 mg·L^−1^·day^−1^ (Fig. 6G). After MB treatment, the treated water in the cultures had P-PO_4_^3−^ concentrations below 1 mg·L^−1^, with removal percentages reaching 80% (Fig. 6H).

Table 3 includes a summary of the composition of treated water after the MB treatment and MFCs + MB treatment.

**Table 3:** Composition of treated water after the MB treatment and MFCs + MB treatment

Parameter	Control	5% PS		10% PS		15% PS	
		MB	MFCs + MB	MB	MFCs + MB	MB	MFCs + MB
COD (mg·L^−1^)	72.03 ± 5.10	515.33 ± 18.88	400.67 ± 48.01	725.33 ± 195.00	558.00 ± 32.00	900.67 ± 140.30	766.67 ± 32.13
N-NH_4_^+^ (mg·L^−1^)	0.4 ± 0.3	0.8 ± 0.1	0.3 ± 0.6	0.5 ± 0.1	0.3 ± 0.6	0.4 ± 0.2	0.0 ± 0.0
N-NO_2_^−^ (mg·L^−1^)	0.0 ± 0.0	0.024 ± 0.002	0.011 ± 0.005	0.043 ± 0.011	0.025 ± 0.006	0.050 ± 0.017	0.026 ± 0.002
N-NO_3_^−^ (mg·L^−1^)	< LOD	< LOD	< LOD	< LOD	< LOD	< LOD	< LOD
TN (mg·L^−1^)	15.33 ± 0.76	32.33 ± 1.53	21.33 ± 2.52	52.33 ± 3.51	33.00 ± 9.64	52 ± 9.64	38 ± 2
P-PO_4_^−3^ (mg·L^−1^)	0.20 ± 0.111	0.53 ± 0.06	0.37 ± 0.06	1.03 ± 0.15	0.57 ± 0.40	1.93 ± 1.097	0.90 ± 0.10

^*^PS, Pig Slurry; *MB*, microalgae-bacteria; *MFCs*, Microbial Fuel Cells; < *LOD*, Limit of detection

### Overall nutrient recovery by MFC-MB

**Fig. 7:**
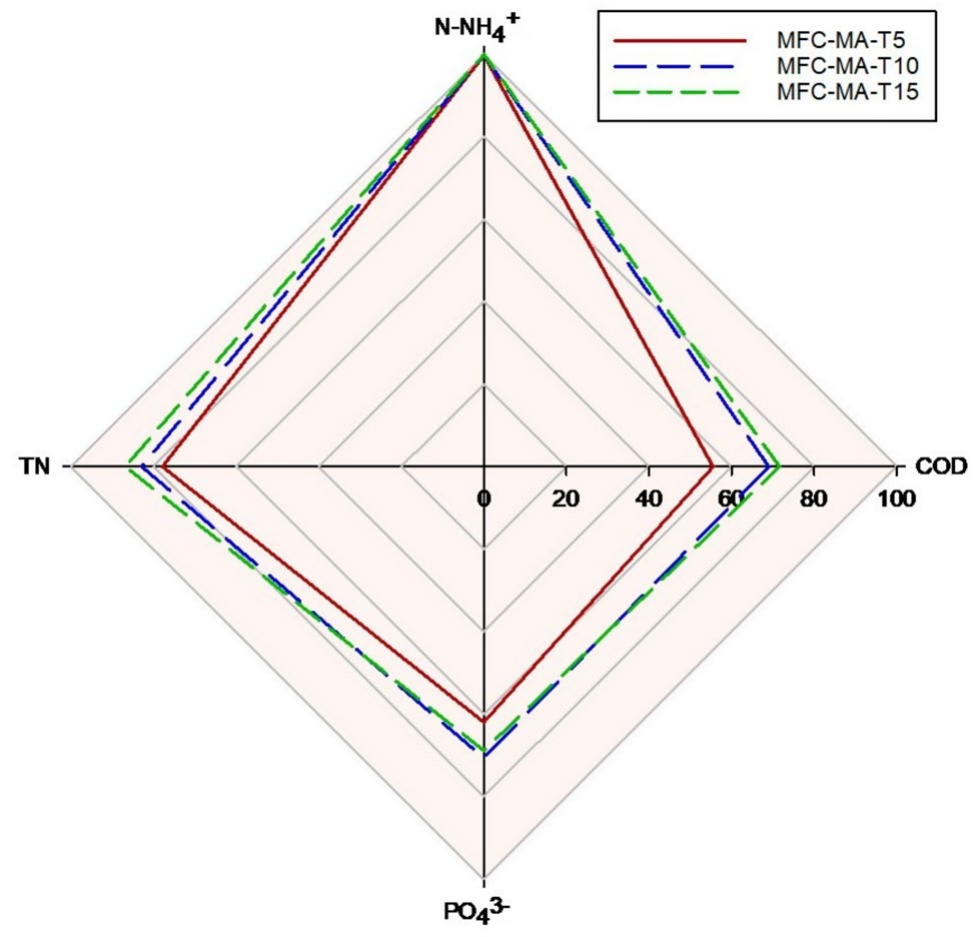
Overall recovery yields of the main nutrients (COD, N-NH_4_^+^, TN and P-PO4^3^⁻) present in PS using a treatment combining MFCs + MB (B)

After completing the MB experiments, the study was assessed based on the efficiency of nutrient recovery from PS. Figure 7 shows the recovery rates for COD, N-NH_4_^+^, TN, and P-PO_4_^3−^ for each treatment in the combined experiment MFCs-MB. As can be seen, all treatments demonstrated high effectiveness in completely recovering N-NH_4_^+^, with no significant differences (*p* > 0.05). Related to P-PO_4_^3−^ recovery, it ranged from 61 to 70% in the combined treatment. COD recovery varied between 56 and 72%, which is notably higher than the 44% to 67% range observed in the MB-only treatment. Regarding TN, the recovery rates ranged from 78 to 87%.

## Discussion

### Bio-electricity generation by MFCs

MFCs offer a sustainable alternative to conventional wastewater treatment by simultaneously generating electricity and removing organic pollutants (Bird et al. 2022). PC analysis is a key method for assessing MFC electrical efficiency and identifying potential losses that limit performance, namely activation, ohmic, and mass transport losses (Hassan et al. 2021). These insights support the optimization of operational and design parameters to improve overall system efficiency (Taufemback et al. 2024). In Fig. 2A, the PC shows voltage as a function of current density. At low current densities, the curve exhibits an exponential shape, attributed to activation losses (Logan et al. 2006). Activation losses occur at low current densities due to the energy required for electron transfer at the electrode surfaces (Abrevaya et al. 2015). In this stage, electrons generated within the electroactive biofilm encounter multiple barriers before reaching the anode or cathode, and some are lost through neutralization or alternative electron acceptors. At moderate current densities, voltage declines linearly because of ohmic losses stemming from the electrical resistance of electrodes, interfaces, and the electrolyte–membrane system. Minimizing these losses is essential to achieving high power densities. In single-chamber MFCs, the cathode’s exposure to air provides direct O_2_ access, reducing mass transport limitations observed at high current densities. These losses, mainly linked to limited oxidant supply or product accumulation, can destabilize system performance and lower voltage output under high-load conditions. The OCV values obtained in this study are quite similar, with Iniesta-López et al. 2023, reporting an OCV of 430 mV, using PS as a fuel and [N_8,8,8,1_^+^][Cl^−^] as IL. In this study, PIM was based on the IL [N_8–10,8–10,8–10,1_^+^][Cl^−^] instead of [N_8,8,8,1_^+^][Cl^−^]. The proposed IL, [N_8–10,8–10,8–10,1_^+^][Cl^−^], presents three chains ranging from 8 to 10 C and a methyl group. This change leads to a less pure IL, but it considerably lowers the production expenses. The greater variability in chain length in [N_8–10,8–10,8–10,1_^+^][Cl^−^] might affect its structural consistency, but its cost advantage could make it an appealing option for industrial applications where cost-efficiency is essential.

On the other side, the power curve (57.27 ± 10.99 mW·m⁻^2^) is lower than the 118 mW·m⁻^2^ reported in a previous work using PS as a fuel and [N_8,8,8,1_^+^][Cl^−^] as IL (Iniesta-López et al. 2023). These results are in line with the conclusions of other studies on IL membranes (Koók et al. 2017), which demonstrated that MFC performance is significantly dependent on the specific IL used, as the substitution of the [N_8,8,8,1_^+^][Cl^−^] with [N_8–10,8–10,8–10,1_^+^][Cl^−^] in this study could have a comparable impact to the differences reported between [Bmim][NTf_2_] and [C_6_mim][PF_6_], confirming that the cationic/anionic structure of the ionic liquid is a critical factor in optimizing MFC performance. However, other operational factors are crucial to compare MFCs performance such as the COD concentration. In this study, a fuel with high COD concentration was used, and there is a known relationship between COD and electricity production. Previous studies hypothesized that increasing the Organic Load Rate (OLR) can reduce electricity production in MFCs for several reasons. Higher OLRs can lead to membrane fouling, which reduces voltage output. Additionally, higher OLRs may cause the accumulation of volatile fatty acids (VFAs) (He et al. 2015), hindering electricity production, and increase methanogenic activity, instead of electrogenic bacteria. MFCs typically reach peak voltage at an optimal OLR, with further increases diminishing performance (Ye et al. 2019). On the other side, compared to the study by Iniesta-López et al. 2023, which reported an internal resistance of 1093 Ω, the value obtained in this study is higher. This increased internal resistance may account for the low power output of the MFCs, as greater resistance hinders electrical flow and reduces overall cell performance.

In the context of the search for alternatives to traditional Nafion®-based membranes, previous studies have shown that [N_8–10,8–10,8–10,1_^+^][Cl^−^], based membranes can achieve between 40 and 50% of the maximum power density generated by Nafion®-based systems (Iniesta-López et al. 2025). However, the power density obtained here is within the wide performance range reported for systems using various separator technologies. Comprehensive literature reviews comparing the performance of supported ionic liquid membranes with standard Nafion membranes and other candidates have consistently shown that the maximum power curve values are largely comparable across all types of separators. Statistical analyses often find no significant difference in the membrane categories, suggesting that factors like internal resistance, OLR management, and overall cell architecture play a dominant role in determining the final power output (Bakonyi et al. 2020).

While Nafion® continues to be the gold standard for PEMs due to its high performance, its use is increasingly questioned because of its environmental persistence. The strong carbon–fluorine bonds in its structure confer extreme chemical stability, resulting in resistance to degradation and long-term accumulation in soils, aquatic systems, and even human tissues. IL-based membranes, due to their advantageous physicochemical properties such as near-zero vapor pressure, high ionic conductivity, and enhanced ion selectivity, represent a promising alternative to traditional PEMs for MFC applications. Although they may exhibit lower environmental persistence compared to perfluorinated membranes, further research on their leaching behaviour and ecotoxicological impact is necessary to substantiate any claims regarding environmental compatibility or sustainability (Sharma et al. 2024).

### Nutrients removal by MFCs

A COD removal efficiency of 56.24 ± 0.38% was achieved, representing a reduction of more than half of the initial COD. Previous reviews have reported COD removal efficiencies in MFCs treating different types of wastewaters ranging from 13.5% to 97%, with 21 studies achieving over 75% removal, thus meeting EU discharge standards. In particular, pig wastewater has been shown to yield lower removal rates (40–60%) due to the presence of more resistant pollutants, which is consistent with the performance observed in this study (Bird et al. 2022).

The COD removal achieved here was relatively high considering the elevated organic load of the PS. This indicates that the IL-based MFCs maintained efficient degradation under high-strength conditions. A positive correlation between influent COD and removal efficiency suggests that system performance depends on substrate concentration. The effect of hydraulic retention time (HRT) remained unclear, though shorter HRTs may be feasible without major efficiency losses, supporting process optimization and cost reduction (Bird et al. 2022).

The high suspended solids (SS) reduction observed (80.74 ± 2.93%) aligns with previous pilot-scale studies reporting SS removal efficiencies between 50 and 95%, depending on reactor configuration and wastewater characteristics. This performance likely results from the combined action of physical entrapment, microbial degradation, and electrochemical oxidation mechanisms operating synergistically within the MFC (Jiang et al. 2011; Zhang et al. 2013; Linares et al. 2019).

Overall, these findings confirm the dual capacity of MFCs to remove organic matter and solids while producing bioelectricity, although optimization of internal resistance and operational stability will be required to enhance COD removal beyond current levels.

### Microalgae-bacteria production

The use of microalgae for wastewater treatment presents notable benefits, such as low energy consumption, greenhouse gas reduction, and resource recovery. However, its efficiency in treating PS is considerably lower compared to municipal wastewater. This is due to the specific properties of PS, which include toxic heavy metals as well as high concentration of N–NH₄^+^. Additionally, light transmission is reduced because of increased turbidity and color caused by SS and dissolved organic matter, which limits microalgal photosynthesis and further obstructs nutrient recycling (Ciardi et al. 2022c; Yu et al. 2024).

In this study, the use of untreated PS diluted at 5–15% allowed biomass productivities comparable to the control culture, although only the 15% PS treatments reached similar cell densities. This suggests that the higher productivity observed in untreated PS may result from bacterial proliferation stimulated by the higher organic matter content. In mixed cultures, bacteria are primarily responsible for organic carbon consumption (Su et al. 2022b). PS pretreated with MFCs showed slightly lower productivity at low dilutions, likely due to the reduction of available nutrients during electrochemical processing. Nevertheless, at higher dilutions (15%), growth parameters approached those of the control, indicating that pretreated PS remains a suitable medium for microalgae cultivation. Trace element scarcity (e.g., Cu, Mn, Mo) in PS may also limit microalgal performance, even when macronutrients such as N are abundant. Moreover, some essential elements, such as P, were present in very low concentrations. This limitation could be mitigated by nutrient supplementation or co-culture strategies enhancing micronutrient bioavailability (Su et al. 2022a).

Taken together, these findings support PS can be viewed as a valuable resource rather than waste. The cooperation between microalgae and bacteria promotes pollutant removal and biomass generation, enabling the production of high value bioproducts within a circular economy framework (Rojo et al. 2023). Recent studies have further demonstrated that microalgae cultivated in waste-derived media, including pig slurry and other wastewater streams, can achieve stable productivity and safe biomass composition, even in the presence of antibiotics or emerging contaminants (Bongiorno et al. 2020; Ruales et al. 2024; Morillas-España et al. 2025).

### Nutrient recovery by microalgae-bacteria consortia

In microalgae-bacteria-based wastewater treatment, MB consortia have proven to be more efficient than pure cultures of bacteria or microalgae for nutrient recovery and treatment. In such systems, N-NO_3_^−^ is actively absorbed by microalgae, reduced to N-NH_4_^+^, and converted into amino acids, while N-NH_4_^+^ is directly taken up and transformed by microalgae and bacteria. P-PO_4_^3−^ is utilized through phosphorylation, and organic carbon is consumed in respiration. Bacteria break down substances unavailable to microalgae, produce CO_2_, and aid in N-NH_4_^+^ oxidation to N-NO_3_^−^. This symbiotic relationship enhances overall nutrient removal and supports stable treatment performance (Yu et al. 2023).

When diluted PS was used as a culture medium, COD removal ranged from moderate to high, with the best performance obtained at higher dilutions. The obtained consumption rates are like previous studies with *S. almeriensis* using fresh PS, which showed that an increase in the COD concentration of the culture medium leads to higher consumption and removal percentages, achieving values of 60% or higher (Ciardi et al. 2022c). The higher COD removal observed in 15% PS treatments suggests that bacterial abundance played a major role in carbon degradation.

Regarding N, no N-NO_3_^−^ or N-NO_2_^−^ was detected in the PS used for the experiment, with the majority of N present as N-NH_4_^+^. The system demonstrated remarkable efficiency, with N-NH_4_^+^ being almost completely removed across all treatments. This outcome aligns with González-Fernández et al. 2011, who reported elimination capacities around 26 mg·L^−1^·day^−1^, subsequently increasing with higher N-NH_4_^+^ loading rates (300–570 mg·L^−1^) using open ponds and PS for algae production.

P is essential for microalgae, involved in energy transfer, cell growth, and forming DNA, RNA, and cell membranes. P removal was also substantial, exceeding 80% in several treatments. Microalgae can directly accumulate polyphosphates and retain them for extended periods, while bacteria release and re-assimilate P dynamically. The coexistence of both groups likely improved overall P recovery compared to conventional bacterial processes (Slocombe et al. 2020; Fallahi et al. 2021).

After characterizing nutrient recovery from diluted PS by MB consortia, the focus shifted to evaluating nutrient consumption and recovery from PS treated with MFC technology. MFC pre-treatment effectively reduced COD while generating energy, though subsequent COD removal by MB was lower, likely due to the decreased fraction of readily biodegradable organic matter. PS biodegradability can vary from 0 to 80% due to swine manure management practices like shed cleaning or waste storage conditions (González et al. 2008). In contrast, N and P recovery remained consistently high, demonstrating the adaptability of MB consortia to the modified effluent composition.

From a practical perspective, these results suggest that when biomass production is prioritized, higher PS dilutions (e.g., 15%) are preferable at least under batch conditions. In continuous systems, high ammonium concentrations may exert inhibitory effects on microalgal activity, as reported elsewhere (Ciardi et al. 2022a). Whereas MFC pre-treatment provides a cleaner effluent for downstream reuse when treatment is the main objective. This flexibility makes the integrated process adaptable to different operational goals.

### Overall nutrient recovery by MFC-MB

Currently, there are different national and European regulations regarding the discharge and reuse of wastewater. Specifically, there are several regulations and directives at the European levels (Directive 91/676/EEC, UE 2019/1009) that govern the discharge and reuse of PS due to its high nutrient content, such as N and P, which can cause serious environmental issues if not properly managed. However, the high variability in PS composition, linked to animal growth stage, feed, and farm management, poses challenges for process standardization n (Antezana et al. 2016; Ciardi et al. 2022c, a, b). Therefore, the development of flexible and robust treatment systems is essential.

The results of this study demonstrated that the combined MFC-MB treatment not only enabled the complete valorization of PS through biomass production comparable to that achieved with chemical fertilizers and green energy generation but also achieved a high recovery rate of the primary nutrients present in PS, along with a cleaner effluent. Nutrient recovery is particularly significant for COD, with the possibility of recovering more than 10% additional COD when applying a pre-treatment of PS with the MFC technology based on ionic liquid membranes.

### Implications of the proposed technique

The integration of MFC and MB technologies offers a promising approach for waste management in intensive livestock production, with the potential to enable simultaneous bioenergy generation, nutrient recovery, and wastewater treatment. This combined system could contribute to the objectives of circular bioeconomy initiatives and align with current European directives on resource efficiency. The use of IL-based PIMs as an alternative to perfluorinated membranes may reduce reliance on environmentally persistent compounds and lower operational costs, although further evaluation of their environmental behaviour is still needed. In particular, future studies should focus on assessing the long-term stability of ILs within the membrane matrix, their potential for reuse, and any associated leaching or ecotoxicological effects. Challenges also remain regarding the scalability and durability of IL-based MFCs, as well as the optimization of MB consortia under variable pig slurry compositions. Pilot-scale trials will be essential to determine the robustness of the integrated system under real operating conditions. In the long term, this approach could support the development of decentralized treatment units for small to medium-sized farms, promoting local resource recovery and reuse.

## Conclusions

The integration of MFCs and MB systems proved effective for treating and recovering nutrients from PS while simultaneously generating bioenergy and biomass. MFCs equipped with the IL membrane [N_8–10,8–10,8–10,1_^+^][Cl^−^] achieved over 50% COD removal and a power output of 57.27 ± 10.99 mW·m⁻^2^, confirming their potential as sustainable alternatives to perfluorinated membranes. MB cultivation using both untreated PS (15%) and MFC-pretreated effluents produced biomass productivities and cell densities comparable to the control. The combined MFC–MB system enabled N–NH₄⁺ recovery rates approaching 100% across treatments, with P-PO₄^3−^ recovery rates between 65 and 85%.

Future work should aim to enhance MFC performance through PS dilution or pre-treatment, and to minimize water demand in MB processes via effluent recirculation. As results were obtained under controlled laboratory conditions, pilot-scale validation is essential to confirm long-term stability and feasibility for industrial implementation.

## Data Availability

Data supporting these reported results are available from the corresponding author upon reasonable request, where applicable.
